# Ca^2+^‐binding protein centrin 4 is a novel binding partner of rat Eag1 K^+^ channels

**DOI:** 10.1002/2211-5463.12045

**Published:** 2016-03-07

**Authors:** Po‐Hao Hsu, Yi‐Chih Chiu, Ting‐Feng Lin, Chung‐Jiuan Jeng

**Affiliations:** ^1^ Institute of Anatomy and Cell BiologySchool of MedicineNational Yang‐Ming UniversityTaipeiTaiwan; ^2^ Brain Research CenterNational Yang‐Ming UniversityTaipeiTaiwan

**Keywords:** EF‐hand calcium‐binding protein, immunoprecipitation, neuron, voltage‐gated K^+^ channel, yeast two‐hybrid screening

## Abstract

Eag1 is neuron‐specific K^+^ channel abundantly expressed in the brain and retina. Subcellular localization and physiological analyses in neurons reveal that Eag1 may participate in Ca^2+^‐signaling processes in the synapse. Here, we searched for rat Eag1 (rEag1)‐binding proteins that may contribute to Ca^2+^ regulation of the K^+^ channel. Yeast two‐hybrid screening identified centrin 4, a member of the centrin family of Ca^2+^‐binding proteins. GST pull‐down and immunoprecipitation assays in brain and retina lysates confirm the interaction of centrin with rEag1 in neurons. Centrin 4 binds to rEag1 in the absence of Ca^2+^. Raising Ca^2+^ concentration enhances the association efficiency of centrin 4 and rEag1, and is required for the suppression of rEag1 currents by centrin 4. Altogether, our data suggest that centrin 4 is a novel binding partner that may contribute to Ca^2+^ regulation of rEag1 in neurons.

**Abbreviations**
CAD: carboxyl assembly domain
CaM: calmodulin
Cetn: centrin
CNBHD: cyclic nucleotide‐binding homology domain
DMSO: dimethyl sulfoxide
Eag: *ether‐à‐go‐go*
GFP: green fluorescent protein
GST: glutathione *S*‐transferase
HEK: human embryonic kidney
PMSF: phenylmethylsulfonyl fluoride
PSD: postsynaptic density
rEag: rat Eag
SPM: synaptosomal

The ether‐à‐go‐go potassium (K^+^) channel family, a member of the voltage‐gated K^+^ (Kv) channel superfamily, comprises three subfamilies: Eag (Kv10), Erg (Kv11), and Elk (Kv12) 1. Eag is neuron‐specific K^+^ channel that encompasses two isoforms, Eag1 (Kv10.1) and Eag2 (Kv10.2), both of which are expressed in a wide variety of different regions in the brain 2, 3, 4, 5. Previous studies from our lab have demonstrated that the subcellular localization of rat Eag1 (rEag1) channels in neurons is characterized by a distinct punctate immunofluorescence pattern that reflects the localization of the K^+^ channel at presynaptic axon terminals and postsynaptic dendritic spines 6, 7, 8.

Activity‐dependent calcium (Ca^2+^) influxes play a pivotal role in regulating neurotransmitter release and synaptic signaling 9, 10. Given its unique subcellular localization pattern in neurons, Eag1 is likely to participate in Ca^2+^ signaling processes in the synapse. In line with this notion, Eag1 appears to modulate presynaptic Ca^2+^ influx and neurotransmitter release during high‐frequency neuronal firing 11. Moreover, calmodulin (CaM) and Ca^2+^/CaM‐dependent protein kinase II, two Ca^2+^‐binding/activated molecules widely implicated in synaptic signaling mechanisms 12, 13, serve as dynamic binding partners of mammalian Eag1 and *Drosophila* Eag channels, respectively 14, 15, 16, 17; for example, CaM binds to and inhibits the function of mammalian Eag1 in a Ca^2+^‐dependent manner. Furthermore, the acidic Ca^2+^‐binding protein S100B seems to interact with human Eag1 channel via a similar Ca^2+^‐dependent mechanism 18.

Here, we aim to search for additional Ca^2+^‐binding proteins of Eag1 K^+^ channels. Our biochemical analyses suggest that the Ca^2+^‐binding protein centrin 4 is a novel binding partner of rEag1. Unlike CaM and S100B, however, centrin 4 appears to associate with rEag1 in the absence of Ca^2+^.

## Materials and methods

### Yeast two‐hybrid screening

The DupLEX‐A yeast two‐hybrid system (OriGene, Rockville, MD, USA) was employed to screen a rat brain cDNA library as previously reported 19. Briefly, the cDNA portion corresponding to rEag1 carboxyl‐terminal region was subcloned into the yeast expression plasmid pGilda, which was used as the bait to screen the library. Positive colonies were further selected by β‐galactosidase assay, followed by plasmid DNA extraction. Candidate cDNA clones were screened by PCR with pJG4‐5‐specific primers and subject to sequence analyses.

### DNA transfection

The cDNA clones used for transfection include rEag1 in pcDNA3 (Invitrogen, Carlsbad, CA, USA) or pEGFP (Clontech, Mountain View, CA, USA), rEag2 in pEGFP, rat centrin 3 and 4 in pcDNA3.1‐Myc, rat calmodulin in pcDNA3.1‐Myc, and human centrin 2 in pEGFP (Addgene 29559; Cambridge, MA, USA) or p‐CMV‐Tag3 (Stratagene, San Diego, CA, USA). Transient transfection of human embryonic kidney (HEK) 293T cells was performed as previously described 20. Transfected cells were maintained at 37 °C for 48 h before being processed for biochemical experiments.

### Immunoprecipitation and immunoblotting

Immunoprecipitation and immunoblotting were performed as described previously 6, 20. In brief, transfected cells were solubilized in ice‐cold IP buffer [20 mm Tris–HCl, pH 7.4, 150 mm NaCl, 10 mm Na_2_HPO_4_, 1% Triton X‐100, 0.5% Na‐deoxycholate, 0.1% SDS, 1 mm EDTA, and 1 mm phenylmethylsulfonyl fluoride (PMSF)]. Where indicated, 2 mm EGTA or 2 mm CaCl_2_ was added in lieu of EDTA. Solubilized lysates were incubated for 16 h at 4 °C with protein A and G sepharose beads (GE Healthcare Biosciences, Marlborough, MA, USA) precoated with the indicated rabbit and mouse antibodies, respectively. Protein samples were separated on 7.5–15% SDS/PAGE, transferred to nitrocellulose membranes, followed by immunoblotting. For detecting centrin signal, membranes were fixed with 0.2% glutaraldehyde prior to primary antibody incubation. Input represents 5% of the total protein used for immunoprecipitation. The antibodies include mouse anti‐β‐actin (Sigma, St. Louis, MO, USA), mouse anti‐centrin (Millipore, Billerica, MA , USA), rabbit anti‐rEag1 (Alomone, Jerusalem, Israel), rabbit anti‐GFP (Abcam, Eugene, OR, USA), mouse anti‐GST (Sigma), mouse IgG (Sigma), mouse anti‐Myc (clone 9E10), mouse anti‐PSD‐95 (NeuroMab, Davis, CA, USA), and mouse anti‐synaptophysin. Results shown are representative of at least three independent experiments.

### Glutathione *S*‐transferase pull‐down assay

Glutathione *S*‐transferase (GST) fusion proteins were produced and purified by following the manufacturer's instruction (Stratagene) as previously reported 19. Briefly, cDNAs encoding indicated regions of rEag1 or centrin 4 were subcloned into the pGEX vector (GE Healthcare Biosciences) and expressed in the *E. coli* strain BL21. The lysates of IPTG‐induced bacteria were incubated with glutathione‐agarose beads (Sigma) that bind GST fusion proteins. GST fusion protein‐coated beads (4–8 μg) were subsequently incubated overnight with appropriate HEK293T cell lysates at 4 °C, and eluted by boiling for 5 min in the Laemmli sample buffer.

### Subcellular fractionation of rat brains

Rat brain homogenates were prepared as described previously 6, 19. All animal procedures were approved by the Institutional Animal Care and Use Committee (IACUC) of National Yang‐Ming University. In brief, adult rat forebrains were homogenized in buffer H1 [(in mm) 320 sucrose, 1 NaHCO_3_, 0.5 CaCl_2_, 0.1 PMSF] containing a cocktail of protease inhibitors (Roche) and centrifuged at 1400 ***g*** to remove nuclei and other large debris (P1). The S1 fraction was subject to centrifugation at 13 800 ***g*** to obtain a crude synaptosome fraction (P2). The pellet was resuspended in buffer H2 (320 mm sucrose, 1 mm NaHCO_3_) and layered onto the top of the discontinuous sucrose density gradient by using 0.85, 1.0, and 1.2 m sucrose layers. The gradient was centrifuged at 65 000 ***g*** for 2 h in a Beckman Instruments SW‐28 rotor. The synaptosomal (SPM) fraction was recovered from the 1.0–1.2 m sucrose interface, followed by extraction with ice‐cold 0.5% Triton X‐100/50 mm Tris–HCl (pH 7.9) for 15 min and centrifugation at 32 000 ***g*** for 45 min to obtain the postsynaptic density (PSD) I pellet. The pellet was resuspended and further extracted with 0.5% Triton X‐100/50 mm Tris–HCl (pH 7.9), followed by centrifugation at 200 000 ***g*** for 45 min to obtain the PSD II pellet. Protein concentration was determined by the BCA protein assay kit (Thermo Fisher Scientific, Waltham, MA, USA).

### Retinal lysate preparation

For retinal lysate preparation, rat retinas were dissected and collected in the lysis buffer (150 mm NaCl, 20 mm Tris, pH7.4, 0.1% sodium dodecyl sulfate, 1% Triton X‐100, 0.25% deoxycholate, 1 mm EDTA) with protease inhibitors and 1 mm PMSF. Retinas were homogenized by dounce homogenizer and centrifuged at 15 000 ***g*** for 15 min at 4 °C. The supernatants were used for GST pull‐down experiments.

### Electrophysiology

Whole‐cell patch clamp in HEK293T cells was performed at 24–48 h post‐transfection as previously reported 19. Patch electrodes with a resistance of ~ 4 MΩ were filled with a solution containing (in mm) 140 KCl, 1 MgCl2, 10 EGTA, 10 HEPES, pH 7.2. External bath solution comprised (in mm) 140 NaCl, 5 KCl, 1 CaCl_2_, and 10 HEPES, pH 7.2. Conventional two‐electrode voltage‐clamp recordings were performed in *Xenopus* oocytes as described previously 20. Briefly, capped cRNA was transcribed *in vitro* from linearized cDNA, and 41.4 nl of cRNA (0.1 μg·μL^−^ ^1^) was injected into each oocyte. Two days after cRNA injection, oocytes were functionally assayed in Ringer solution [(in mm): 115 NaCl, 3 KCl, 1.8 CaCl_2_, 10 HEPES, pH 7.2]. Niflumic acid (0.5 mm) was added to minimize the contribution of endogenous Ca^2+^‐activated Cl^−^ currents. A23187 was dissolved in dimethyl sulfoxide (DMSO) as stock solution for storage at −20 °C, which was diluted to 1 μM in the Ringer solution on the day of experiment. Alternatively, identical amount of DMSO was added to the Ringer solution as the control solution. Voltage‐clamp protocols were applied with the pclamp 8.2/9.0 software (Molecular Devices, Sunnyvale, CA, USA). Data were acquired with OC‐725C oocyte clamp (Warner) (for *Xenopus* oocytes) or Axopatch 200B amplifier (Molecular Devices) (for HEK293T cells), followed by digitization at 10 kHz with the Digidata 1320A/1322A system (Molecular Devices). Data were filtered at 1 kHz and passive membrane properties were compensated with the ‐P/4 leak subtraction method by using the pclamp 8.2/9.0 software. All recordings were performed at room temperature (20–22 °C).

## Results

### Identification of centrin 4 as a binding partner of rEag1

To search for rEag1‐interacting Ca^2+^‐binding proteins, we carried out yeast two‐hybrid screening of a rat brain cDNA library by using the intracellular carboxyl‐terminal region of the K^+^ channel as the bait. Among the 102 positive hits, 5 clones are CaM, and 7 clones correspond to centrin 4, which is an isoform of the centrin protein family that, like CaM, belongs to the superfamily of EF‐hand Ca^2+^‐binding proteins 21, 22, 23, 24.

**Figure 1:**
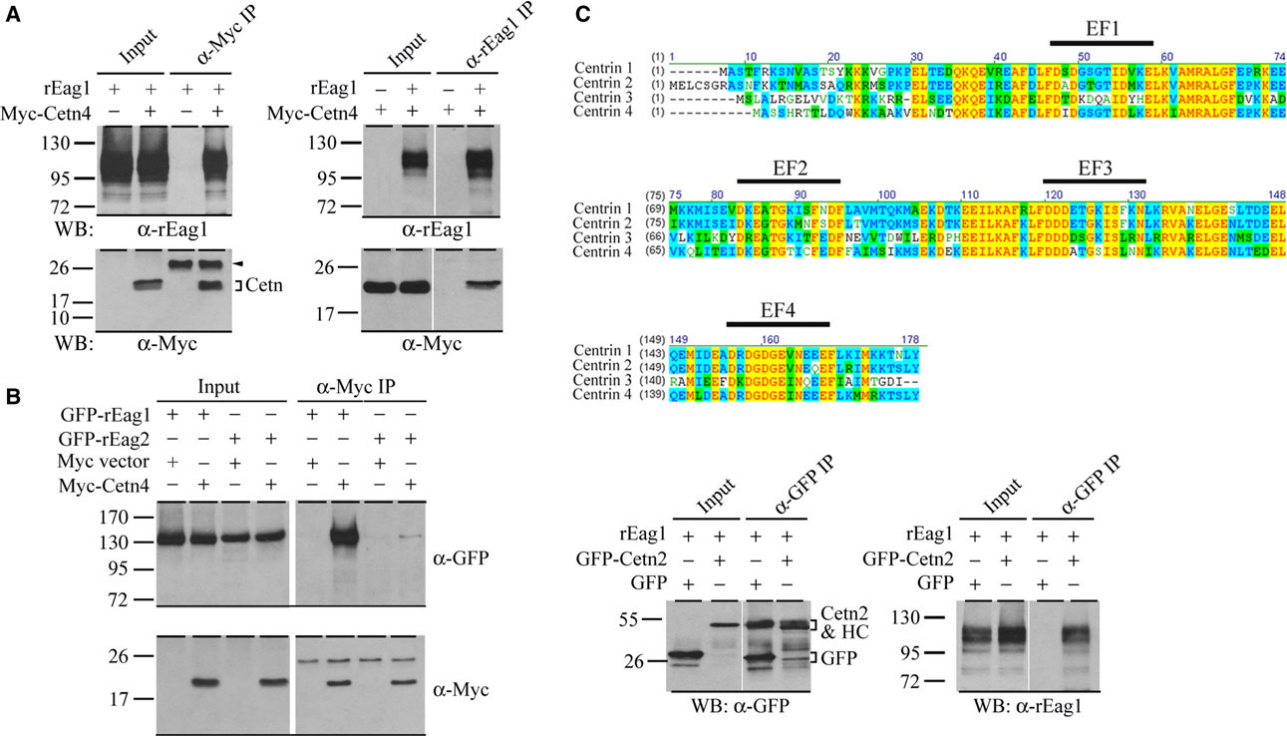
Centrin 4 and rEag1 coexist in the same protein complex in HEK293T cells. (A) Coimmunoprecipitation of Myc‐centrin 4 (Myc‐Cetn4) and rEag1. Coexpression with the Myc vector was used as the control experiment. The molecular weight markers (in kiloDaltons, kDa) are labeled to the left. Cell lysates were immunoprecipitated (IP) with the anti‐Myc *(*α‐Myc, *left panel)* or anti‐rEag1 *(*α‐rEag1, *right panel)* antibody, followed by immunoblotting (WB) with the indicated antibodies (α‐rEag1 or α‐Myc). Corresponding expression levels of rEag1 and Myc‐centrin 4 in the lysates are shown in the *Input* lane. The predicted molecular weight of Myc‐centrin 4 is about 21 kDa. Arrow head denotes the IgG light chain arising from immunoprecipitation with the anti‐Myc antibody. (B) Coimmunoprecipitation of GFP‐rEag1, but not GFP‐rEag2, and Myc‐centrin 4. Cell lysates were immunoprecipitated with the anti‐Myc antibody, followed by immunoblotting with the anti‐GFP or anti‐Myc antibody. (C) *(Upper panel)* Sequence alignment of the four centrin isoforms by using the Vector NTI software. EF refers to the putative EF‐hand Ca^2+^‐binding motif. *(Lower panel)* Coimmunoprecipitation of GFP‐centrin 2 and rEag1. Coexpression with the GFP vector was used as the control. Cell lysates were immunoprecipitated with the anti‐GFP antibody, followed by immunoblotting with the anti‐GFP or anti‐rEag1 antibody. The predicted molecular weight of GFP‐centrin 2 is about 47 kDa, which results in an electrophoretic mobility indistinguishable from that of the IgG heavy chain (HC) of the anti‐GFP antibody.

To confirm the association of centrin 4 with rEag1, we performed immunoprecipitation experiments by transiently coexpressing Myc‐tagged centrin 4 with rEag1 in HEK293T cells. As illustrated in Fig. 1A, upon immunoprecipitating Myc‐centrin 4 with the anti‐Myc antibody, significant rEag1 signal is recognized; conversely, immunoprecipitation with the anti‐rEag1 antibody leads to efficient detection of Myc‐centrin 4. The specificity of our immunoprecipitation experiments was verified by the finding that Myc‐centrin 4 is coimmunoprecipitated with GFP‐rEag1, but not GFP‐rEag2 (Fig. 1B). Together these observations indicate that centrin 4 indeed coexist in the same protein complex with rEag1.

The centrin protein family comprises four isoforms: centrin 1‐4 (Fig. 1C). Interestingly, in addition to centrin 4, our yeast two‐hybrid screening also identifies centrin 3. Moreover, by coexpressing rEag1 with GFP‐tagged centrin 2, which is closely related to centrin 4 22, we found that centrin 2 is readily coimmunoprecipitated with rEag1 (Fig. 1C). These data suggest that centrin 2 and 3 may also serve as binding partners of rEag1.

### Interaction of centrin with rEag1 in neurons

**Figure 2:**
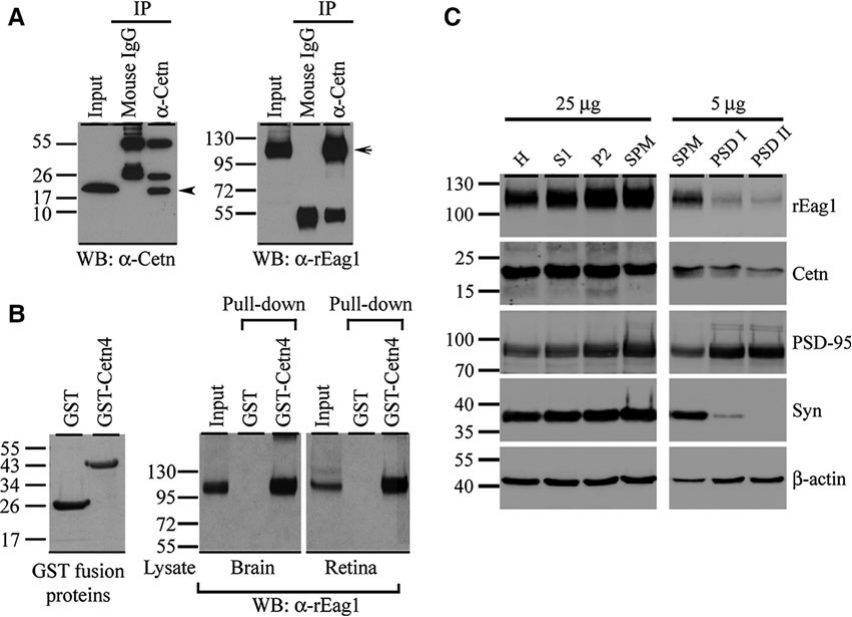
Association of centrin with rEag1 in neurons. (A) Coimmunoprecipitation of centrin (Cetn) and rEag1. Rat forebrain homogenates were immunoprecipitated with the mouse IgG or the anti‐centrin antibody, followed by immunoblotting with the anti‐centrin or anti‐rEag1 antibody. Arrow and arrow head denote IgG heavy and light chains, respectively. (B) *(Left panel)* Coomassie blue staining of GST and GST‐centrin 4 fusion proteins. *(Right panel)* GST pull‐down assay of brain and retina lysates with the GST or the GST‐centrin 4 fusion proteins, followed by immunoblotting with the anti‐rEag1 antibody. (C) Sucrose fractionation of rat brain homogenates: homogenate (H), soluble fraction (S1), crude membrane fraction (P2), and synaptosomal fraction (SPM), and the two SPM subfractions PSD I and PSD II. The left panel (*25* μ*g*) represents the primary fractionation profile, whereas the right panel (*5* μ*g*) depicts the further enrichment pattern in the synaptosomal subfractions. All fractions were subject to immunoblotting analyses with the indicated antibodies. Synaptophysin (Syn) and PSD‐95 were used as pre‐ and postsynaptic markers, respectively. Twenty‐five and 5 μg refer to the amount of total protein loaded in each lane.

Both centrin and Eag1 are abundantly expressed in the brain 2, 22, 25 and the retina 8, 24, 26. To determine whether these two proteins are binding partners in native cells, rat forebrain homogenates were subject to immunoprecipitation analyses with an anti‐centrin antibody recognizing all four centrin isoforms, followed by immunoblotting with the anti‐rEag1 antibody. As demonstrated in Fig. 2A, rEag1 is effectively coimmunoprecipitated with centrin. Furthermore, GST pull‐down assay with a GST fusion protein encoding centrin 4 (GST‐centrin 4) shows that, in both brain and retina lysates, rEag1 is precipitated by GST‐centrin 4, but not by the GST protein *per se* (Fig. 2B), consistent with the idea that centrin 4 interacts with rEag1 in the brain and the retina. Next we asked whether centrin and rEag1 share overlapping subcellular localization in the brain. By performing sucrose gradient centrifugation of rat forebrain homogenates, we collected the synaptosomal (SPM) fraction and the SPM subfractions PSD I and PSD II that enables to identify proteins localized in presynaptic and/or postsynaptic compartments. As illustrated in Fig. 2C, the presynaptic marker synaptophysin is highly enriched in the SPM fraction and virtually absent in the PSD II fraction; on the contrary, PSD‐95 is highly enriched in both the PSD I and PSD II fractions. Importantly, both centrin and rEag1 are present in the SPM fraction and the SPM subfractions, implying a colocalization in the synapse. Altogether these data provide strong evidence in support of the association of endogenous centrin proteins with rEag1 in neurons.

### Centrin 4 binds to the carboxyl‐terminal region of rEag1

**Figure 3:**
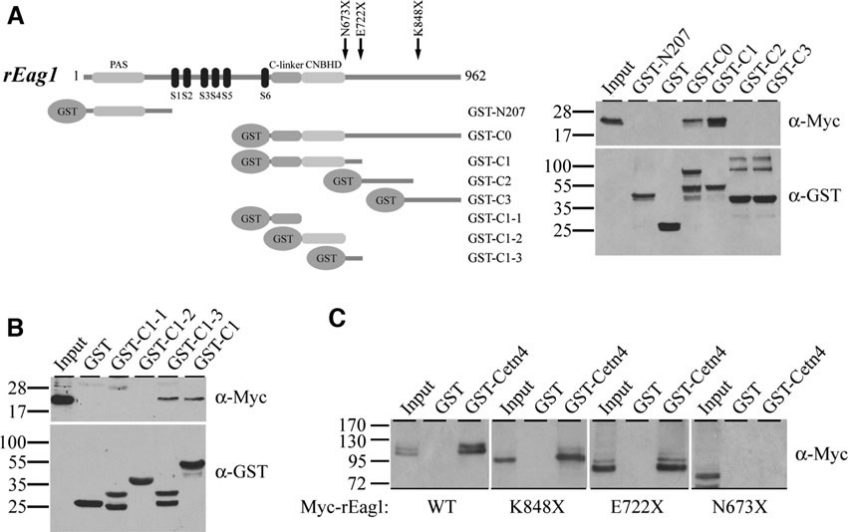
Localization of the centrin 4‐binding domain in rEag1. (A) *(Left panel)* Schematic topology of full‐length rEag1 and various GST‐rEag1 fusion proteins. *(Right panel)* GST pull‐down assay with the indicated GST‐rEag1 amino‐ or carboxyl‐terminal fusion proteins. Lysates from HEK293T cells overexpressing Myc‐centrin 4 were employed for the pull‐down assay, followed by immunoblotting with the anti‐Myc antibody. (B) GST pull‐down of Myc‐centrin 4 with the indicated GST‐rEag1 carboxyl‐terminal fusion proteins. (C) GST‐centrin 4 pull‐down of Myc‐rEag1 wild‐type (WT) or each of the indicated truncation mutants.

To localize the centrin 4‐binding region(s) within rEag1, we generated GST fusion proteins encoding either the amino‐terminal (GST‐N207) or the carboxyl‐terminal (GST‐C0) region of the K^+^ channel. Figure 3A depicts that Myc‐centrin 4 is effectively pulled down by the GST‐rEag1 fusion protein GST‐C0, but not GST‐N207, indicating that centrin 4 primarily interacts with the intracellular carboxyl‐terminal region of rEag1.

rEag1 contains a long intracellular carboxyl‐terminal region spanning over 470 amino acids that can be divided into three structural domains: the carboxyl linker (C‐linker), the cyclic nucleotide‐binding homology domain (CNBHD), and the post‐CNBHD region (Fig. 3A). To further address the structural basis of centrin 4‐rEag1 interaction, we produced three GST fusion proteins encoding specific carboxyl‐terminal sections of rEag1: GST‐C1 (amino acids 493–724; including the C‐linker, the CNBHD, and the initial segment of the post‐CNBHD region), GST‐C2 (amino acids 723–848; the proximal post‐CNBHD region), and GST‐C3 (amino acids 835–962; the distal post‐CNBHD region). Figure 3A illustrates that only the GST‐C1 fusion protein displays significant centrin 4‐binding efficiency, suggesting that the proximal part of rEag1 carboxyl‐terminal region may harbor the major centrin 4‐interacting domain. We, therefore, created three additional GST fusion proteins that encode the C‐linker (GST‐C1‐1: amino acid 493–560), the CNBHD (GST‐C1‐2: amino acids 561–672), or the initial segment of the post‐CNBHD region (GST‐C1‐3: amino acid 673–722). As depicted in Fig. 3B, centrin 4 preferentially binds to GST‐C1‐3. Furthermore, we performed immunoprecipitation experiments of centrin 4 with three different rEag1 carboxyl‐terminal truncation mutants (N673X, E722X, K848X) that were previously characterized in our lab 27. While all of the three rEag1 truncation mutants contain intact C‐linker and CNBHD, only N673X lacks the initial segment of the post‐CNBHD region. Figure 3C illustrates that centrin 4 is effectively coimmunoprecipitated with E722X and K848X, but not N673X. Taken together, these data support the notion that the centrin 4‐binding site(s) may predominantly reside in the initial segment of the post‐CNBHD region in rEag1.

### Ca^2+^ is not required for the interaction of centrin 4 with rEag1

**Figure 4:**
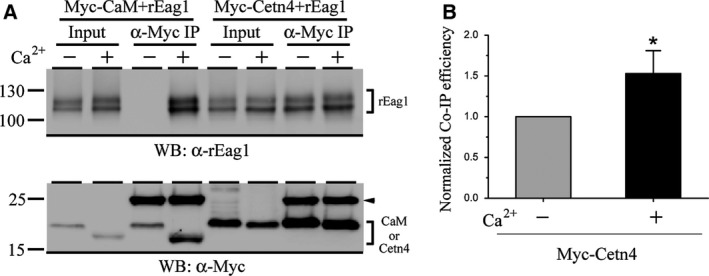
Centrin 4 binds to rEag1 in the absence of Ca^2+^. (A) Coimmunoprecipitation of Myc‐calmodulin (CaM) *(left panel)* or Myc‐centrin 4 (Cetn4) *(right panel)* and rEag1 in HEK293T cells. Immunoprecipitation with the anti‐Myc antibody was performed in the absence (−; 2 mm EGTA) or presence (+) of 2 mm CaCl_2_. The predicted molecular weight of Myc‐CaM is about 17 kDa. Arrow head denotes IgG light chain. (B) Coimmunoprecipitation (Co‐IP) efficiency of Myc‐Cetn4 and rEag1 in the absence or presence of Ca^2+^. Protein signals were subject to densitometric scan and thereafter quantified by using the ImageJ software. Co‐IP efficiency was calculated as the ratio of rEag1 signal to the cognate centrin 4 signal in each IP lane, followed by normalization with respect to the no‐Ca^2+^ control. Both values are presented as mean ± SEM. Asterisk denotes significant difference from the control (*, *t*‐test: *P* < 0.05; *n* = 3).

A single CaM contains four EF‐hand Ca^2+^‐binding motifs that can cooperatively bind four Ca^2+^ 28. By contrast, despite the presence of four EF‐hand Ca^2+^‐binding homology motifs, only the fourth EF‐hand motif in centrin 4 may effectively bind Ca^2+^ 22. Moreover, both CaM and S100B bind to human Eag1 channel in a Ca^2+^‐dependent manner 14, 18. We, therefore, asked whether the interaction between centrin 4 and rEag1 also displays a similar requirement for Ca^2+^. As shown in the left panel of Fig. 4A, the effectiveness of Ca^2+^ concentration change was verified by the prominent electrophoretic mobility shift between Ca^2+^‐free and Ca^2+^‐bound CaM; as expected, coimmunoprecipitation of rEag1 with Myc‐CaM was only observed when 2 mm CaCl_2_ was present. The Ca^2+^‐induced alteration in electrophoretic mobility for Myc‐centrin 4, however, is markedly smaller (Fig. 4A, right panel), consistent with the notion that centrin 4 comprises only one Ca^2+^‐binding site 22. Most importantly, association of rEag1 with Myc‐centrin 4 was detected in the absence and the presence of 2 mm CaCl_2_ (Fig. 4A). Therefore, these data suggest that, unlike CaM and S100B, Ca^2+^‐free centrin 4 may associate with rEag1. Nevertheless, the coimmunoprecipitation efficiency of centrin 4 and rEag1 is enhanced by raising Ca^2+^ concentration (Fig. 4B).

### Centrin 4 reduces rEag1 current amplitude in the presence of Ca^2+^

**Figure 5:**
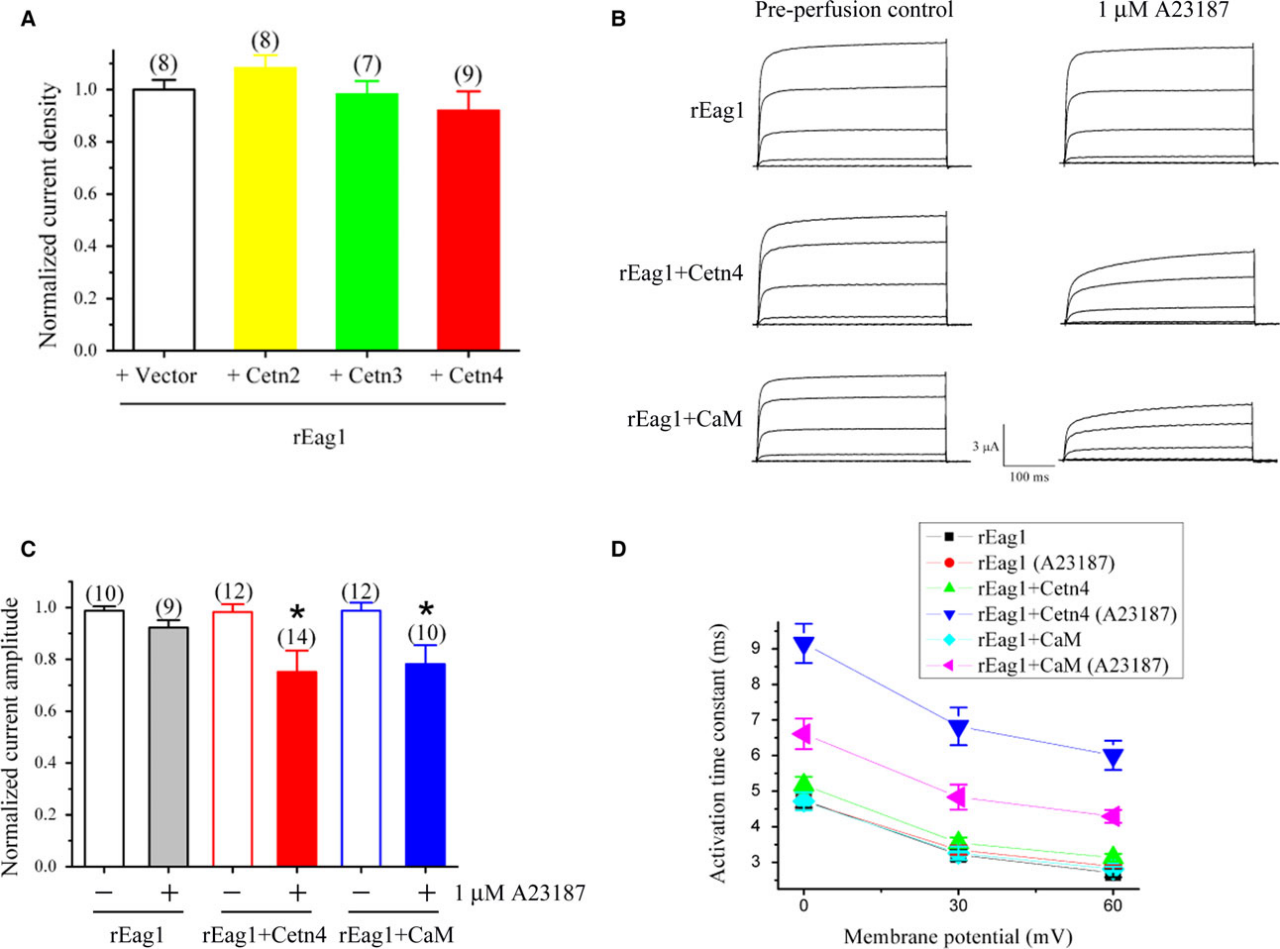
Centrin 4 suppresses rEag1 K^+^ currents in a Ca^2+^‐dependent manner. (A) Lack of effect of centrin coexpression on rEag1 current density. Data were acquired by performing whole‐cell patch clamp recordings in HEK293T cells. rEag1 was coexpressed in the molar ratio 1 : 5 with the Myc vector (Vector), Myc‐centrin2 (Cetn2), Myc‐centrin3 (Cetn3), or Myc‐centrin4 (Cetn4). The mean value for steady‐state rEag1 current density at +60 mV of each centrin coexpression condition was normalized with respect to that of the vector control. (B) Representative K^+^ current traces showing the effect of the Ca^2+^‐ionophore A23187 on rEag1 alone (rEag1), or coexpressing (in the molar ratio 1 : 5) rEag1 with centrin 4 (rEag1 + Cetn4) or calmodulin (rEag1 + CaM). Data were collected by conducting two‐electrode voltage‐clamp recordings in *Xenopus* oocytes From a holding potential at −90 mV, oocytes were subject to a series of six 370‐ms test pulses ranging from −90 mV to +60 mV, in 30‐mV increments. rEag1 K^+^ currents were recorded from the same oocyte before (Preperfusion control) and after the perfusion of 1 μM A23187 in the Ringer solution. The stability of rEag1 current levels during the preperfusion control period was monitored for at least 4 min before the A23187 perfusion was initiated. Upon coexpressing with Cetn4 or CaM, reduction of rEag1 current levels was usually observed at about 2 min after the beginning of A23187 perfusion. The perfusion effect was continuously monitored for at least 10 min. To avoid the influence of potential nonselective effects imposed by the Ca^2+^‐ionophore, the data shown and analyzed here were taken at 2 min after A23187 perfusion was initiated. Passive membrane properties were compensated using the –P/4 leak subtraction method. (C) Coexpression with Cetn4 or CaM manifests Ca^2+^‐dependent reduction in rEag1 currents. For each of the three rEag1 expression groups, oocytes were perfused with the DMSO control solution (−) or the 1 μM A23187 solution (+). For each perfusion condition of a given rEag1 expression group, steady‐state K^+^ current amplitudes at +60 mV were normalized with respect to the mean values derived from the corresponding preperfusion control. Mean current amplitudes recorded on the same day were normalized with respect to those of the corresponding control condition. Normalized data collected from different days were pooled together for statistical analyses. Asterisks denote significant difference from the corresponding DMSO control (*, *t*‐test: *P* < 0.05). (D) Coexpression with Cetn4 or CaM manifests Ca^2+^‐dependent deceleration of rEag1 activation kinetics. Activation time constants at indicated potentials were obtained from single exponential fits to the late rising phase of rEag1 K^+^ currents. All values are shown as mean ± SEM.

Having characterized the physical interaction between centrin 4 and rEag1, we moved on to address the functional effect of centrin 4 on rEag1 channels. Coexpression with centrin 2, 3, or 4 (up to the molar ratio 1 : 5) fails to discernibly affect the functional expression of rEag1 in HEK293T cells (Fig. 5A), suggesting that, like CaM, the low intracellular Ca^2+^ concentration is insufficient to support the potential rEag1‐regulating effect of centrin 4. Identical results were obtained when we repeated the same functional assay by coexpressing centrin 4 with rEag1 in *Xenopus* oocytes (data not shown). We, therefore, attempted to increase intracellular Ca^2+^ concentration by employing the Ca^2+^‐ionophore A23187 that was dissolved in the extracellular Ringer solution containing 1.8 mm CaCl_2_. As demonstrated in Fig. 5B,C, in the absence of centrin 4 or CaM, perfusion of 1 μM A23187 does not appreciably alter rEag1 current level in *Xenopus* oocytes. By contrast, in the presence of centrin 4 or CaM, application of 1 μM A23187, but not the DMSO control solution, leads to a notable reduction in rEag1 K^+^ current amplitude, indicating that both centrin 4 and CaM inhibit the function of rEag1 in a Ca^2+^‐dependent manner. Interestingly, in the presence of enhanced intracellular Ca^2+^ concentration, coexpression with centrin 4 or CaM also notably slows the activation kinetics of rEag1 currents (Fig. 5D).

## Discussion

In this report, we provide a series of different biochemical evidence suggesting that centrin 4, as well as centrin 2 and 3, may interact with rEag1. In vertebrate retinal photoreceptors, all four centrin isoforms are densely expressed in either the basal body complex in the inner segment, or the connecting cilium linking the outer and the inner segments; moreover, centrin forms a Ca^2+^‐dependent protein complex with the G‐protein transducin that is essential for phototransduction 23, 24. Interestingly, previous immunofluorescence data from our lab reveal that rEag1 is also abundantly expressed in the outer and the inner segments of photoreceptors 8. In good agreement with our observation, two splice variants of Eag1 channels have been shown to express in bovine photoreceptors, and may represent the noninactivating K^+^ current contributing to the outward dark current in the inner segment of rod photoreceptors 26. Furthermore, GST pull‐down assay in this study shows that centrin 4 is directly associated with rEag1 in the retina. Taken as a whole, these observations imply that centrin may modulate rEag1 signaling in photoreceptors.

Like Eag1, centrin 2 and 3 are broadly expressed in the brain 22. Using an anti‐centrin antibody that recognizes all four centrin isoforms, we demonstrate here that centrin and rEag1 coexist in the same protein complex in the brain, and that centrin colocalizes with rEag1 in the synapse. Given the unique subcellular localization pattern of rEag1 in neurons, we speculate that, in addition to CaM, centrin 2 and/or 3 are likely to participate in Ca^2+^‐dependent regulations of rEag1 activity at presynaptic and/or postsynaptic sites. The localization of centrin 4 in the brain, however, is restricted to the ependymal cells lining different ventricles and the choroidal cells in the choroid plexus 22. Interestingly, a strong rEag1‐immunostaining signal was also detected in ependymal region of lateral ventricle or subventricular zone that contains neuroblasts 25. Since centrin 4 is found in centrosomes that are involved in cell division 22, it remains to be determined whether centrin 4 and rEag1 may participate in neural differentiation and/or adult neurogenesis in ependymal neuroblasts.

Eag1 channel comprises three distinct CaM‐binding domains: one in the amino‐terminal (amino acids 151–165) and two in the carboxyl‐terminal (amino acids 674–683 and 711–721) regions 14, 15. Using GST fusion proteins encoding specific amino‐ or carboxyl‐terminal sections of rEag1, we discover that the centrin 4‐binding domain mainly resides in the initial segment (amino acids 673–722) of the post‐CNBHD region in rEag1, which seems to overlap with the two carboxyl‐terminal CaM‐binding domains. This finding raises an intriguing possibility that centrin 4 and CaM may share and perhaps compete for the same binding site(s) in the intracellular carboxyl‐terminal region of rEag1.

In direct contrast with CaM, however, Ca^2+^‐free centrin 4 may associate with rEag1. Similar lack of Ca^2+^ requirement has also been reported for CaM binding to small‐conductance Ca^2+^‐activated K^+^ (SK) channels 29, voltage‐gated Ca^2+^ (Ca_V_) channels 30, and KCNQ K^+^ (Kv7) channels 31. Nevertheless, centrin 2 appears to display reversible, Ca^2+^‐dependent self‐association that resembles actin polymerization 32. Moreover, the coimmunoprecipitation efficiency of centrin 4 and rEag1 is increased in the presence of Ca^2+^, consistent with the idea that centrin 4 may go through certain Ca^2+^‐dependent conformational change that in turn augments its association with rEag1. Indeed, using the Ca^2+^‐ionophore A23187, we demonstrate that, similar to SK, Ca_V_, and Kv7 channels, Ca^2+^ is required for the modulation of rEag1 channel function by centrin 4. In other words, despite their striking difference in Ca^2+^ requirement for physical association with the K^+^ channel, both centrin 4 and CaM suppress Eag1 current level in a Ca^2+^‐dependent manner. If we assume that centrin 4 and CaM indeed compete for the same binding site in the carboxyl‐terminal region of rEag1, then it will be important to ascertain how intracellular Ca^2+^ concentration may adjust rEag1 function via this dual Ca^2+^‐senor system.

In conclusion, this study identifies centrin 4 as a novel binding partner of rEag1, which may shed new light on the dynamics of Ca^2+^ regulation of the K^+^ channel. Moreover, our biochemical analyses in neurons highlight the notion that multiple centrin isoforms may contribute to Ca^2+^‐dependent modulation of rEag1 signaling in photoreceptors, as well as in various regions in the brain.

## Author contributions

PHH performed yeast two‐hybrid screening, mutagenesis and subcloning, and biochemical analyses. YCC conducted biochemical and subcellular fractionation analyses. TFL carried out electrophysiological experiments and data analyses. CJJ wrote the manuscript with inputs from the co‐authors.
